# An exploratory study on the influence of ^60^Co irradiation on the volatile organic components and antibacterial activity of *Artemisia argyi* Folium

**DOI:** 10.3389/fchem.2026.2004768

**Published:** 2026-10-29

**Authors:** Ping Xie, Cheng Tian, Yuhuan Liu, Yanxin Dai, Yuqing Liu, Dan Huang, Liang Cao

**Affiliations:** 1 State Key Laboratory of Chinese Medicine Powder and Medicine Innovation in Hunan (Incubation), Academy of Chinese Medical Sciences, Hunan Provincial Key Laboratory of Prevention and Treatment of Depressive, Hunan University of Chinese Medicine, Changsha, China; 2 Dong Ethnic Medicine Research Laboratory of Hunan Province, Hunan University of Medicine, Huaihua, China; 3 Hunan Province Sino-US International Joint Research Center for Therapeutic Drugs of Senile Degenerative Diseases, Hunan University of Chinese Medicine, Changsha, China; 4 Hunan Engineering Technology Research Center for Bioactive Substance Discovery of Chinese Medicine, Changsha, China

**Keywords:** ^60^Co irradiation, antibacterial activity, Artemisia argyi Folium, gas chromatography-ion mobility spectrometry, volatile organic components

## Abstract

**Background/Objectives:**

*Artemisia argyi* Folium, renowned as the “king of herbs” and one of the oldest medicinal plants in China, is popular worldwide. However, it is highly susceptible to contamination by bacteria, molds, and insect eggs during harvesting, drying, and processing. ^60^Co irradiation is currently applied as an effective method. This study aimed to assess the impact of ^60^Co irradiation at 0, 3, 6, and 9 kGy on the volatile organic component (VOC) profile and antibacterial activity of *A. argyi* Folium essential oil (EO).

**Methods:**

GC-IMS, antibacterial assays, and multivariate statistical analysis to analyze the variation patterns of VOCs in the EO of *Artemisia Argyi* Folium, as well as corresponding changes in antibacterial activity, before and after ^60^Co irradiation treatment at various intensities.

**Results:**

A total of 111 headspace derived VOCs were tentatively identified. The VOC profiles of the 3.0 kGy and 6.0 kGy groups were similar, while 9.0 kGy irradiation was associated with significant changes. Based on agar diffusion assay results, the 6.0 kGy group exhibited the largest inhibition zone against *Escherichia coli*, whereas the 3.0 kGy group produced the maximum inhibition zone against MRSA. Correlation analysis revealed that α-Phellandrene was strongly negatively correlated with MRSA IC_50_, suggesting it may act as a candidate marker associated with the antibacterial activity against MRSA.

**Conclusion:**

The results indicate that, within the tested dose range, lower irradiation doses (3.0 and 6.0 kGy) may be more favorable than 9.0 kGy for preserving the VOC profile, higher doses (6.0 and 9.0 kGy) improved the MIC and MBC values against MRSA. It provided a reliable reference for rational irradiation dose selection for *A. argyi* Folium processing and quality preservation.

## 1 Introduction

*Artemisia argyi* Folium is the dried leaf of *A. argyi* Lévl. et Vant., a well-known medicinal-edible plant rich in phytochemicals with diverse pharmacological activities (Xie et al., 2025). Its volatile organic compounds (VOCs), flavonoids and polysaccharides contribute to its antibacterial, immunomodulatory and antioxidant properties (Chen et al., 2024; Wang et al., 2024). VOCs are the primary active compounds determining its medicinal potency and quality evaluation (Gao and Lu, 2026).

As a commonly used dried-herb raw material, *A. argyi* Folium is vulnerable to contamination by bacteria, fungi and insect eggs during harvesting and post-harvest processing. Pathogenic microorganisms cause herbal deterioration and potential health risks, severely compromising the safety and shelf-life quality of the herb. Effective microbial decontamination is therefore essential to guarantee its pharmaceutical quality. Conventional decontamination strategies include thermal treatment, chemical disinfection and irradiation. Thermal processing is economical and residue-free but unsuitable for herbs containing thermolabile volatiles (Baky et al., 2024). Chemical disinfection, such as ethylene-oxide fumigation, bears risks of reagent residues and variable efficacy (Lee et al., 2025). By comparison, ^60^Co irradiation exhibits high penetration, residue-free properties and excellent microbe-elimination effects, and is frequently applied at 3.0–6.0 kGy for herbal materials (Lei et al., 2023; Zou et al., 2025).

Headspace volatile profiling frequently employs gas chromatography-ion mobility spectrometry (GC-IMS) due to its high sensitivity and fast analysis (Zheng et al., 2025; Wesoly et al., 2024; Jin et al., 2023). Multivariate statistics including principal component analysis (PCA) and partial least-squares discriminant analysis (PLS-DA) enable discrimination of differential volatile compounds (Chen et al., 2023; Li et al., 2024; Ma et al., 2025). Our group previously reported irradiation-induced VOC changes in several medicinal plants, yet those studies only focused on chemical profiles and neglected corresponding biological activity alterations (Lei et al., 2023; Xiang et al., 2023; Zou et al., 2025; He et al., 2023).

Accordingly, this work adopted GC-IMS to characterize dose-dependent headspace VOC variations of hydrodistilled *A. argyi* Folium essential oil (EO), combined with systematic antibacterial bioassays. We aimed to unravel the relationships among irradiation dose, VOC compounds and antibacterial capacity, to provide references for selecting rational irradiation doses for *A. argyi* Folium processing. Few reports have simultaneously addressed irradiation-modified headspace VOCs and antibacterial performance of its EO. These findings could provide a reference for rational dose-selection in ^60^Co irradiation processing of *A. argyi* Folium.

## 2 Materials and methods

### 2.1 Materials

*Artemisia argyi* Folium was collected from three geographically distinct sites (27.573843°N 110.152359°E, 27.588642°N 110.186379°E, 27.623749°N 110.206379°E) in Huaihua, Hunan Province, China in date of 15 June 2025. Species identification was performed by Prof. Zhi Wang from Hunan University of Chinese Medicine, based on macroscopic and microscopic morphological characteristics, in reference to the Pharmacopoeia of the People’s Republic of China (2025 edition). A representative voucher specimen (HNUCM2025-AY001) of *A. argyi* was deposited in the herbarium of Hunan University of Chinese Medicine. Specimens were stored under dark conditions at 20 °C ± 2 °C with relative humidity ≤45%. *Escherichia coli* (ATCC 25922) and Methicillin-resistant *Staphylococcus aureus* (MRSA) (ATCC 43300) were used; both are preserved at the Hunan Provincial Key Laboratory of Synthetic Biology of Traditional Chinese Medicine, Hunan University of Medicine. Strains were maintained in 20% (v/v) glycerol suspensions at −80 °C and sub-cultured no more than three times prior to experiments to guarantee phenotypic stability.

### 2.2 ^60^Co radiation

Dried *A. argyi* Folium samples (hot-air drying, at 50 °C with an air velocity of 0.4 m/s for 10 h) from three independent biological batches were divided evenly into four groups: AY-01, AY-02, AY-03, and AY-04. Each subsample was sealed in a polyethylene sterile bag and irradiated with ^60^Co γ-rays at a professional irradiation facility (Hunan Radiological Technology Application Research Center, Changsha, China). The dose ranges were 0 kGy (control group), 3.0 kGy, 6.0 kGy, and 9.0 kGy. The activity of the radiation source was 2.96 × 10^16^ Bq (Hunan Institute of Nuclear Agriculture and Aerospace Breeding, Changsha, China). To reach the target absorbed doses of 0 kGy (sham-irradiated control), 3.0 kGy, 6.0 kGy, and 9.0 kGy, the corresponding exposure durations were calculated as 0 h, 3.75 h, 7.5 h, and 11.25 h, respectively. For each dose group, the three independent biological batches were separately packaged and irradiated in parallel under identical experimental conditions. During irradiation, all samples were placed horizontally on a polymethyl methacrylate (PMMA) sample holder to achieve homogeneous radiation exposure from all directions. Actual absorbed doses of each sample group were validated by placing calibrated alanine dosimeters at the geometric center of representative sample bags; the measured values were within ±5% of the target doses. All irradiation treatments were carried out at ambient temperature (22 °C ± 2 °C) and relative humidity of 50% ± 10%. Within 24 h after irradiation, samples were stored at 4 °C in darkness for subsequent experiments.

### 2.3 Essential oil extraction

The EO was extracted by hydrodistillation using a Clevenger-type apparatus according to the Pharmacopoeia of the People’s Republic of China (2025 edition). In brief, the dried *A. argyi* Folium sample (500 g) was placed in a round-bottomed flask with 1,600 mL of distilled deionized water, heated to boiling, and then maintained at a gentle boil for 6 h. The EO was co-evaporated with water vapor and collected in the condenser. The oil layer was separated, sealed in a sample tube, and stored in the dark at 4 °C until analysis. The extraction yields for AY-01, AY-02, AY-03, and AY-04 were 2.1% ± 0.2%, 1.9% ± 0.1%, 2.0% ± 0.2%, and 2.0% ± 0.1% (w/w), respectively (mean ± SD, n = 3). Each irradiation group was extracted with three independent biological replicates. Prolonged hydrodistillation may trigger thermal transformation and hydrolysis, altering the intrinsic volatile profile of raw herbal leaves. In the present study, the EO of *A. argyi* Folium specifically refers to the oil obtained by 6 h hydrodistillation. Unless otherwise specified, VOCs mentioned hereinafter denote headspace detectable volatile compounds of this hydrodistilled EO.

### 2.4 GC–IMS analysis

The following instruments and equipment were used: FlavourSpec® Gas-Phase Ion Mobility Spectrometer (G.A.S., Dortmund, Germany); CTC-PAL 3 Static Headspace Autosampler (CTC Analytics AG, Zwingen, Switzerland); and VOCal data processing software (version 0.4.10, G.A.S., Dortmund, Germany).

Each sample was prepared by transferring 10 µL of EO into a 20 mL headspace vial, followed by incubation at 80 °C for 20 min. For each group, analysis was performed in triplicate, with one injection per sample.

Headspace sampling, rather than direct-liquid injection, was adopted for GC-IMS analysis, because non-volatile residues present in essential oil may contaminate the IMS drift tube. Nevertheless, headspace analysis preferentially captures high-vapor-pressure compounds and does not reflect the full chemical profile of EO.

The headspace sampling conditions were as follows: Incubation temperature: 80 °C; incubation time: 20 min; injection volume: 100 μL; splitless injection mode; incubation agitation speed: 500 r/min; injection needle temperature: 85 °C.

The following GC conditions were applied: column: MXT-WAX fused silica capillary column (30 m × 0.53 mm, 1.0 µm) (Restek Corporation, USA); column temperature: 60 °C; carrier gas: high purity nitrogen (purity ≥99.999%); total run time: 60 min; injector temperature: 80 °C; and program: initial flow rate of 2.0 mL/min held for 2 min, linearly increased to 10.0 mL/min over 8 min, linearly increased to 100.0 mL/min over 10 min, and held for 40 min.

The IMS conditions were as follows: ionization source: tritium (^3^H); drift tube length: 53 mm; electric field strength: 500 V/cm; temperature: 45 °C; drift gas: high purity nitrogen (≥99.999%) at a flow rate of 75.0 mL/min; positive ion mode.

Mixed standards of six ketones (2-butanone, 2-pentanone, 2-hexanone, 2-heptanone, 2-octanone, and 2-nonanone) were analyzed to establish the correlation between retention time and retention index. The retention indices of target compounds were calculated according to their respective retention times. Qualitative analysis of target analytes was accomplished by matching the obtained data with the built-in GC retention index database (NIST 2020) and IMS drift time database embedded in the VOCal software. As a static headspace technique, GC-IMS detects volatile compounds released from EO instead of directly analyzing the liquid-phase composition of the EO. Peak intensities correspond merely to relative signal responses and are not suitable for absolute-quantitative comparison across different analytes. Therefore, all comparisons in the present study are semiquantitative and describe relative changes acquired under identical analytical conditions. For conciseness, these headspace-released volatile compounds originating from EO are hereinafter designated as VOCs throughout this manuscript.

### 2.5 Determination of antibacterial activity

The test strains were inoculated onto solid LB medium (Tryptone: 10 g/L; yeast extract: 5 g/L; sodium chloride: 10 g/L; agar: 15 g/L; pH = 7.0) using a sterile inoculating loop and cultured in an incubator at 37 °C for 1 day. This process was repeated for three successive passages. A single colony was then selected and subjected to culture in liquid LB medium. The bacterial concentration in the liquid LB medium was adjusted to 1 × 10^6^ CFU/mL for subsequent usage.

#### 2.5.1 Reagents and instruments

Blank susceptibility discs (φ6 mm) were purchased from Bikeman Biology; Tween-80 (analytical grade), tryptone, yeast extract, sodium chloride, and other reagents were obtained from Macklin Reagent Co., Ltd.

An autoclave (YXO-100SП, Shanghai Xunbo Industrial Co., Ltd.), biochemical incubator (SPX-250-Z, Shanghai Xunbo Industrial Co., Ltd.), clean bench (AlphGClean1300, Lixin Instrument Shanghai Co., Ltd.), and oscillating incubator (ZQZY-C8V, Shanghai Zhichu Instrument Co., Ltd.) were used.

#### 2.5.2 Determination of antibacterial activity

Antibacterial activity was assessed using the disc diffusion method. A fresh bacterial suspension adjusted to 0.5 McFarland turbidity was evenly spread onto solid LB medium. Each sterile blank susceptibility disc (φ6 mm) was impregnated with 5 μL of EO, while sterile water served as a blank control. Vancomycin (100 μg/mL) was used as the positive control for MRSA, whereas kanamycin sulfate (100 μg/mL) served as the positive control for *E. coli*, with 5 μL of corresponding antibiotic solution loaded onto each disc. All assays were performed in triplicate. The plates were immediately sealed and incubated at 37 °C for 24 h, after which the antibacterial zone of inhibition was observed.

#### 2.5.3 Determination of MIC, MBC, and IC_50_

A 96-well plate was used with 0.1% Tween 80 as an emulsifier. A solvent control group containing 0.1% Tween-80 together with bacterial suspension was included to exclude the antibacterial interference of the emulsifier. Different concentrations of *A. argyi* Folium EO (AY-01----AY-04) were prepared, with final concentrations of 10%, 5%, 2.5%, 1.25%, 0.625%, 0.313%, 0.156%, and 0.078%. Subsequently, 100 μL of diluted bacterial suspension was added to each well, resulting in a final bacterial concentration of 10^5^ CFU per well. A growth control well was set up without any drug, containing only 100 μL of LB medium and 100 μL of bacterial suspension, to verify bacterial viability. Another control well containing only 200 μL of LB medium was included to check for medium contamination. All assays were performed in triplicate. The plates were incubated at 37 °C for 24 h.

For MBC determination, 50 μL of bacterial suspension from each well was spread onto solid LB medium and incubated at 37 °C for 24 h. MBC was defined as the lowest EO concentration yielding no bacterial colonies after agar plating.

MIC was defined as the lowest essential oil concentration at which no visible bacterial growth was observed in the liquid wells after incubation. In the initial screen, clear wells were observed at 1.25% for *E. coli* and 0.156% for MRSA. To more precisely determine the MIC values, a refined concentration series (1.2%, 1.0%, 0.8%, 0.6%, 0.4%, 0.2%, 0.1%, and 0.05%) was subsequently tested using the same procedure.

A series of AY-01-AY-04 dilutions in concentration of 4.0‰, 3.0‰, 2.0‰, 1.5‰, 0.75‰, 0.37‰, 0.187‰, 0.0937‰, 0.0468‰, 0.0234‰, 0.0117‰, 0.0058‰, 0.0029‰, and 0.0015‰ were prepared to test the IC_50_ for *E. coli* and MRSA, using the same method as for the MIC and MBC. After 24 h incubation, the optical density (OD) of each well was measured at 600 nm using a microplate reader. The bacterial inhibition rate was calculated according to the following formula: Inhibition rate (%) = [(ODgrowth control − ODsample)/(ODgrowth control − ODblank control)] × 100%. Non-linear regression was performed in GraphPad Prism (Version 8.4.2) to fit the dose-response curve and calculate the IC_50_ values.

### 2.6 Statistical analysis

Analyses of three-dimensional (3D) spectra, two-dimensional (2D) spectra, and fingerprints for the VOCs were performed using the VOCal data processing software (version 0.4.10) by G.A.S. (Dortmund, Germany). Raw GC-IMS peak intensity matrices were exported from VOCal software after background subtraction and peak alignment. The data matrix was subjected to sum-normalization and then exported in CSV format for subsequent PCA, PLS-DA and hierarchical cluster analysis (CA). Principal component analysis (PCA) was performed using OmicShare Tools. Partial Least Squares Discriminant Analysis (PLS-DA) was performed using SIMCA (Version 14.1, Umetrics, Sweden). The model validity was evaluated via 200-fold cross-validation, and variable importance in projection (VIP) values were calculated for variable screening, with VIP >1 set as the threshold for differential marker selection. Hierarchical cluster analysis (CA) and cluster heatmaps were generated using the Bioinformatics online platform (https://bioinformatics.com.cn/). Pearson correlation analysis was conducted and visualized using the ChiPlot online platform (https://chiplot.online/), with statistical significance defined at p < 0.05 and p < 0.01.

## 3 Results

### 3.1 Analysis of GC-IMS

#### 3.1.1 Comparison of headspace VOC differences in *Artemisia argyi* Folium Subjected to different irradiation doses

The VOCs from *A. argyi* Folium sterilized with different ^60^Co irradiation doses were analyzed using GC-IMS 3D spectra (as shown in Figure 1A). In the 3D spectrum, the X-axis represents the ion drift time, the Y-axis gas chromatography retention time, and the Z-axis peak intensity, with peak height reflecting relative signal response of each VOC. The 2D topographic plot (shown in Figure 1B) shows the GC retention time as the ordinate and the relative drift time as the abscissa. The signal peak at the abscissa 1.0 corresponds to the reactant ion peak (RIP), and all signal peaks were normalized against the RIP peak. Colors ranging from blue to red indicate increasing peak intensities from low to high. Results showed differences among the four sample groups (AY-01, AY-02, AY-03, and AY-04) in terms of characteristic peak numbers, positions, and signal intensities. Furthermore, using AY-01 as a reference, subtraction analysis was performed on the other three sample groups to obtain differential GC-IMS spectra (shown in Figure 1C), with white indicating no difference, red indicating a higher signal intensity compared to the reference, and blue indicating a lower signal intensity. Comprehensive analysis of the 3D spectra, 2D topographic plot, and differential GC-IMS spectra showed that the VOCs from *A. argyi* Folium differed following different ^60^Co irradiation treatments.

**FIGURE 1:**
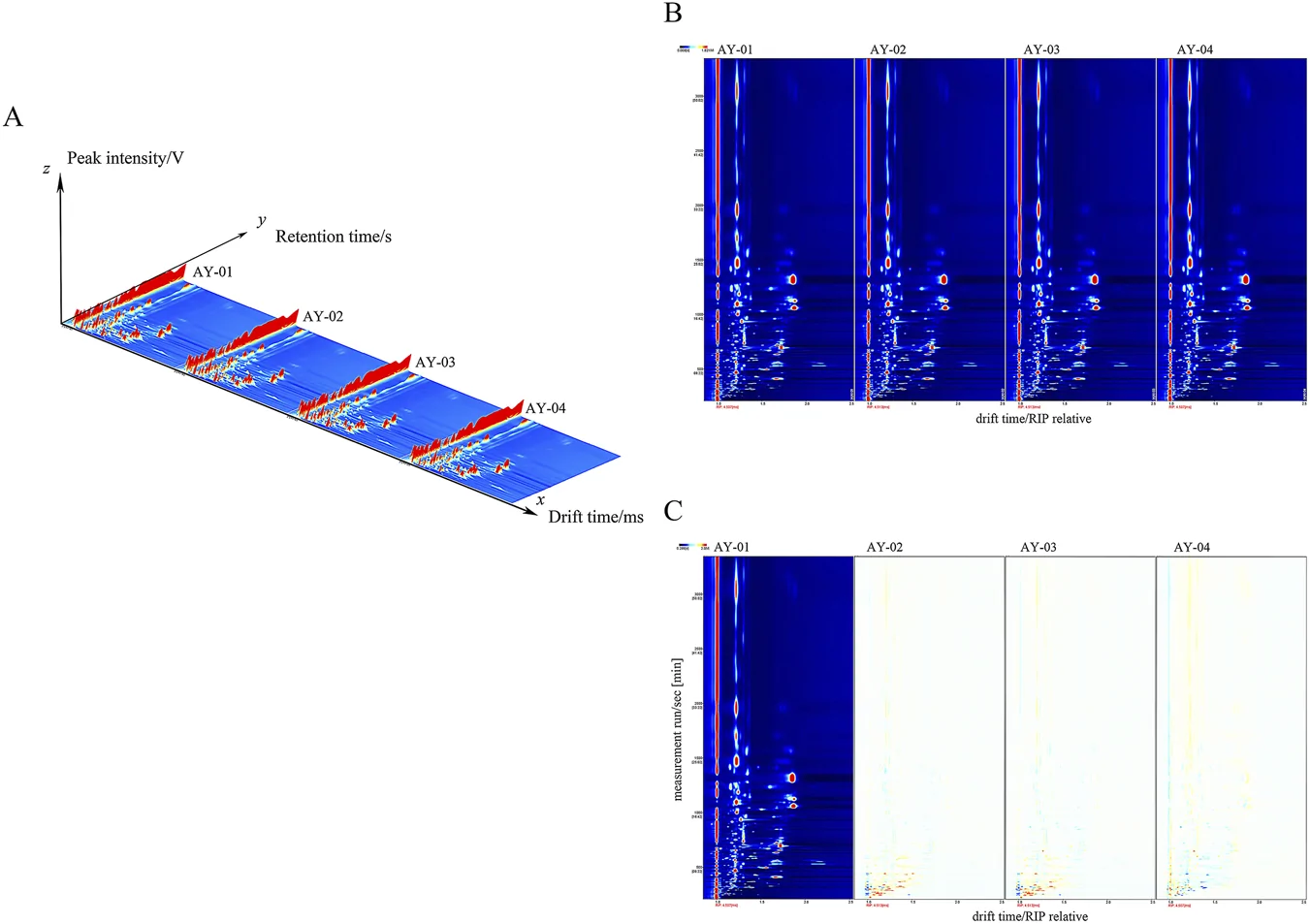
3D GC-IMS spectra of headspace VOCs of hydrodistilled EO from *Artemisia argyi* Folium under different irradiation doses **(A)**; 2D topographic plot of headspace VOCs of hydrodistilled EO from *Artemisia argyi* Folium under different irradiation doses **(B)**; Differential GC-IMS spectra of headspace VOCs of hydrodistilled EO from *Artemisia argyi* Folium under different irradiation doses **(C)**.

#### 3.1.2 Qualitative analysis of headspace VOCs of hydrodistilled EO from *Artemisia argyi* Folium by GC-IMS

In total, 111 VOCs were tentatively identified across the four sample groups, including 21 alcohols, 21 aldehydes, 19 ketones, 14 ethers, 14 heterocycles, 12 terpenes, 5 aromatic hydrocarbons, 3 sulfur-containing compounds, and 2 acids. Specific details of these VOCs are provided in Table 1. The raw peak intensity data for all identified VOCs are provided in Supplementary Table S1.

**TABLE 1:** Detailed list of VOCs.

No	Compound	CAS	Formula	MW	RI	Rt (s)	Dt (RIPrel)
1	Carvone	99-49-0	C_10_H_14_O	150.2	1916.8	3,082.922	1.30405
2	Borneol-M	507-70-0	C_10_H_18_O	154.3	1906.1	3,012.581	1.22256
	Borneol-D	507-70-0	C_20_H_36_O_2_	308.5	1906.1	3,012.581	1.88372
3	α-Terpineol	98-55-5	C_10_H_18_O	154.3	1831.4	2,563.481	1.21886
4	4-Terpinenol-M	562-74-3	C_10_H_18_O	154.3	1700.0	1929.577	1.22000
	4-Terpinenol-D	562-74-3	C_20_H_36_O_2_	308.5	1700.8	1932.886	1.72604
5	d-Camphor-M	464-49-3	C_10_H_16_O	152.2	1,599.3	1,552.499	1.34730
	d-Camphor-D	464-49-3	C_20_H_32_O_2_	304.5	1,599.7	1,553.891	1.84473
6	Isomenthone-M	491-07-6	C_10_H_18_O	154.3	1,523.3	1,317.228	1.34898
	Isomenthone-D	491-07-6	C_20_H_36_O_2_	308.5	1,523.8	1,318.839	1.84481
7	Benzaldehyde-M	100-52-7	C_7_H_6_O	106.1	1,556.1	1,413.915	1.15802
	Benzaldehyde-D	100-52-7	C_14_H_12_O_2_	212.2	1,556.2	1,414.504	1.47569
8	Acetic acid	64-19-7	C_2_H_4_O_2_	60.1	1,503.0	1,260.827	1.06086
9	Furfural	98-01-1	C_5_H_4_O_2_	96.1	1,500.1	1,252.770	1.09436
10	α,p-Dimethylstyrene	1195-32-0	C_10_H_12_	132.2	1,455.3	1,137.341	1.17147
11	1-Hexanol-M	111-27-3	C_6_H_14_O	102.2	1,377.5	961.238	1.33443
	1-Hexanol-D	111-27-3	C_12_H_28_O_2_	204.3	1,377.9	962.118	1.64700
12	6-Methyl-5-hepten-2-one	110-93-0	C_8_H_14_O	126.2	1,355.6	916.930	1.17848
13	cis-3-Hexenyl acetate	3681-71-8	C_8_H_14_O_2_	142.2	1,328.4	864.500	1.30059
14	1-Hydroxy-2-propanone	116-09-6	C_3_H_6_O_2_	74.1	1,320.8	850.469	1.07696
15	Octanal-M	124-13-0	C_8_H_16_O	128.2	1,307.8	826.838	1.41535
	Octanal-D	124-13-0	C_16_H_32_O_2_	256.4	1,307.6	826.571	1.81937
16	Terpinolene	586-62-9	C_10_H_16_	136.2	1,298.1	809.854	1.21820
17	Hexyl acetate	142-92-7	C_8_H_16_O_2_	144.2	1,291.1	798.038	1.38593
18	1-Pentanol-M	71-41-0	C_5_H_12_O	88.1	1,272.1	767.659	1.25450
	1-Pentanol-D	71-41-0	C_10_H_24_O_2_	176.3	1,272.6	768.513	1.51872
19	γ-Terpinene-M	99-85-4	C_10_H_16_	136.2	1,261.8	751.756	1.21144
	γ-Terpinene-D	99-85-4	C_20_H_32_	272.5	1,261.8	751.756	1.70235
20	(E)-2-Hexenal-M	6728-26-3	C_6_H_10_O	98.1	1,241.4	721.127	1.17842
	(E)-2-Hexenal-D	6728-26-3	C_12_H_20_O_2_	196.3	1,241.5	721.281	1.51373
21	Heptanal	111-71-7	C_7_H_14_O	114.2	1,206.9	672.239	1.35067
22	α-Terpinene-M	99-86-5	C_10_H_16_	136.2	1,195.2	656.336	1.21431
	α-Terpinene-D	99-86-5	C_20_H_32_	272.5	1,195.6	656.925	1.72101
23	Myrcene-M	123-35-3	C_10_H_16_	136.2	1,183.8	633.953	1.21431
	Myrcene-D	123-35-3	C_20_H_32_	272.5	1,184.3	635.131	1.70810
24	p-Xylene	106-42-3	C_8_H_10_	106.2	1,154.4	573.285	1.07938
25	β-Pinene-1	127-91-3	C_10_H_16_	136.2	1,131.3	529.698	1.21431
	β-Pinene-2	127-91-3	C_10_H_16_	136.2	1,131.7	530.287	1.30187
	β-Pinene-3	127-91-3	C_10_H_16_	136.2	1,132.0	530.876	1.64063
	β-Pinene-4	127-91-3	C_10_H_16_	136.2	1,132.3	531.465	2.18753
26	Hexanal-M	66-25-1	C_6_H_12_O	100.2	1,112.5	496.683	1.27735
	Hexanal-D	66-25-1	C_12_H_24_O_2_	200.3	1,112.8	497.151	1.55548
27	Camphene-1	79-92-5	C_10_H_16_	136.2	1,096.2	469.780	1.20959
	Camphene-2	79-92-5	C_10_H_16_	136.2	1,096.5	470.194	1.64385
	Camphene-3	79-92-5	C_10_H_16_	136.2	1,096.5	470.194	2.18461
28	α-Pinene-1	80-56-8	C_10_H_16_	136.2	1,049.8	411.008	1.21650
	α-Pinene-2	80-56-8	C_10_H_16_	136.2	1,049.5	410.594	1.29948
	α-Pinene-3	80-56-8	C_10_H_16_	136.2	1,049.5	410.594	1.66736
29	1-Butanol-M	71-36-3	C_4_H_10_O	74.1	1,167.6	599.741	1.18469
	1-Butanol-D	71-36-3	C_8_H_20_O_2_	148.2	1,167.7	599.943	1.39183
30	3-Methyl-2-pentanone-M	565-61-7	C_6_H_12_O	100.2	1,039.6	399.015	1.17635
	3-Methyl-2-pentanone-D	565-61-7	C_12_H_24_O_2_	200.3	1,039.8	399.273	1.47649
31	1-Penten-3-one	1629-58-9	C_5_H_8_O	84.1	1,026.1	383.827	1.09059
32	Ethanol-M	64-17-5	C_2_H_6_O	46.1	960.9	325.908	1.04112
	Ethanol-D	64-17-5	C_4_H_12_O_2_	92.1	961.6	326.423	1.12577
33	2-Propanol-M	67-63-0	C_3_H_8_O	60.1	949.9	318.186	1.09389
	2-Propanol-D	67-63-0	C_6_H_16_O_2_	120.2	950.6	318.701	1.22032
34	3-Methylbutanal	590-86-3	C_5_H_10_O	86.1	945.4	315.097	1.40173
35	2-Butanone-M	78-93-3	C_4_H_8_O	72.1	931.7	305.830	1.06311
	2-Butanone-D	78-93-3	C_8_H_16_O_2_	144.2	931.7	305.830	1.24671
36	Ethyl acetate	141-78-6	C_4_H_8_O_2_	88.1	914.3	294.421	1.09618
37	2-Methyl-2-propenal-M	78-85-3	C_4_H_6_O	70.1	912.8	293.421	1.04990
	2-Methyl-2-propenal-D	78-85-3	C_8_H_12_O_2_	140.2	912.8	293.421	1.22164
38	Butanal-M	123-72-8	C_4_H_8_O	72.1	907.6	290.135	1.13731
	Butanal-D	123-72-8	C_8_H_16_O_2_	144.2	907.8	290.259	1.28262
39	2-Propenal	107-02-8	C_3_H_4_O	56.1	881.1	273.848	1.06224
40	Methyl acetate	79-20-9	C_3_H_6_O_2_	74.1	864.3	263.991	1.19285
41	Acetone	67-64-1	C_3_H_6_O	58.1	856.6	259.562	1.11572
42	2-Methylpropanal	78-84-2	C_4_H_8_O	72.1	850.5	256.133	1.27923
43	Propanal-M	123-38-6	C_3_H_6_O	58.1	836.8	248.561	1.07047
	Propanal-D	123-38-6	C_6_H_12_O_2_	116.2	836.8	248.561	1.14760
44	Dimethyl sulfide	75-18-3	C_2_H_6_S	62.1	811.9	235.418	0.95426
45	Acetaldehyde	75-07-0	C_2_H_4_O	44.1	786.2	222.560	1.03345
46	Neryl acetate	141-12-8	C_12_H_20_O_2_	196.3	1866.0	2,762.208	1.21051
47	2,3-Butanediol	513-85-9	C_4_H_10_O_2_	90.1	1,677.0	1836.231	1.35373
48	1-Octanol	111-87-5	C_8_H_18_O	130.2	1,666.1	1793.338	1.47730
49	1-Octen-3-ol-M	3391-86-4	C_8_H_16_O	128.2	1,493.0	1,233.819	1.16795
	1-Octen-3-ol-D	3391-86-4	C_16_H_32_O_2_	256.4	1,492.9	1,233.688	1.60840
50	2,6-Dimethyl-3-ethylpyrazine	13925-07-0	C_8_H_12_N_2_	136.2	1,497.5	1,245.959	1.21745
51	2,3-Dimethyl-5-ethylpyrazine	15707-34-3	C_8_H_12_N_2_	136.2	1,471.9	1,178.783	1.24633
52	(Z)-3-Hexenol-M	928-96-1	C_6_H_12_O	100.2	1,410.4	1,032.225	1.24449
	(Z)-3-Hexenol-D	928-96-1	C_12_H_24_O_2_	200.3	1,410.0	1,031.3^60^	1.51238
53	2-Ethyl-5-methylpyrazine	13360-64-0	C_7_H_10_N_2_	122.2	1,404.2	1,018.377	1.19282
54	cis-2-Penten-1-ol	1576-95-0	C_5_H_10_O	86.1	1,344.7	895.472	0.94941
55	(E)-2-Heptenal	18829-55-5	C_7_H_12_O	112.2	1,337.5	881.801	1.25463
56	2,6-Dimethylpyrazine	108-50-9	C_6_H_8_N_2_	108.1	1,331.7	870.747	1.13218
57	2,5-Dimethylpyrazine	123-32-0	C_6_H_8_N_2_	108.1	1,318.1	845.515	1.11026
58	1,2,3-Trimethylbenzene	526-73-8	C_9_H_12_	120.2	1,323.3	855.037	1.13972
59	Ethyl hex-3-enoate	2396-83-0	C_8_H_14_O_2_	142.2	1,325.5	859.192	1.25107
60	1-Octen-3-one-M	4312-99-6	C_8_H_14_O	126.2	1,320.0	849.042	1.28014
	1-Octen-3-one-D	4312-99-6	C_16_H_28_O_2_	252.4	1,320.2	849.366	1.68032
61	Butylbenzene	104-51-8	C_10_H_14_	134.2	1,318.8	846.867	1.22142
62	2-Octanone	111-13-7	C_8_H_16_O	128.2	1,304.8	821.497	1.33780
63	3-Methyl-2-butenal	107-86-8	C_5_H_8_O	84.1	1,223.1	694.772	1.09102
64	Pyrazine	290-37-9	C_4_H_4_N_2_	80.1	1,204.0	668.262	1.04596
65	2-Heptanone	110-43-0	C_7_H_14_O	114.2	1,201.3	664.574	1.26192
66	o-Xylene	95-47-6	C_8_H_10_	106.2	1,179.0	623.668	1.06813
67	Diallyl sulfide	592-88-1	C_6_H_10_S	114.2	1,154.7	573.837	1.33320
68	3-Penten-2-one-M	625-33-2	C_5_H_8_O	84.1	1,125.2	518.684	1.09021
	3-Penten-2-one-D	625-33-2	C_10_H_16_O_2_	168.2	1,125.4	519.106	1.34559
69	2,3-Pentanedione	600-14-6	C_5_H_8_O_2_	100.1	1,065.5	430.016	1.21148
70	Pentanal	110-62-3	C_5_H_10_O	86.1	1,015.0	371.749	1.42022
71	2-Pentanone	107-87-9	C_5_H_10_O	86.1	1,011.9	368.371	1.36774
72	2,3-Butanedione	431-03-8	C_4_H_6_O_2_	86.1	1,009.5	365.838	1.18233
73	4-Methyl-2-pentanone	108-10-1	C_6_H_12_O	100.2	1,029.7	387.793	1.47735
74	2-Pentylfuran	3777-69-3	C_9_H_14_O	138.2	1,252.8	738.145	1.25151
75	4-Methyl-3-penten-2-one-M	141-79-7	C_6_H_10_O	98.1	1,160.1	584.582	1.12450
	4-Methyl-3-penten-2-one-D	141-79-7	C_12_H_20_O_2_	196.3	1,160.2	584.795	1.44714
76	(E)-2-Pentenal	1576-87-0	C_5_H_8_O	84.1	1,157.4	579.220	1.35916
77	2-Methyl-1-propanol	78-83-1	C_4_H_10_O	74.1	1,118.4	506.748	1.17077
78	Citronellol	106-22-9	C_10_H_20_O	156.3	1940.6	3,245.273	1.36069
79	Ethyl 3-hydroxyhexanoate-M	2305-25-1	C_8_H_16_O_3_	160.2	1837.7	2,598.619	1.30444
	Ethyl 3-hydroxyhexanoate-D	2305-25-1	C8H16O_3_	160.2	1838.0	2,600.226	1.85880
80	Acetylpyrazine	22047-25-2	C_6_H_6_N_2_O	122.1	1798.8	2,388.901	1.21289
81	Phenylacetaldehyde	122-78-1	C_8_H_8_O	120.2	1775.6	2,272.117	1.26808
82	5-Methyl-6,7-dihydro-(5H)-cyclopentapyrazine	23747-48-0	C_8_H_10_N_2_	134.2	1770.3	2,246.165	1.22333
83	Citronellyl acetate	150-84-5	C_12_H_22_O_2_	198.3	1855.8	2,702.181	1.47991
84	Linalool-M	78-70-6	C_10_H_18_O	154.3	1,639.1	1,691.902	1.22184
	Linalool-D	78-70-6	C_20_H_36_O_2_	308.5	1,639.1	1,691.902	1.69173
85	Propanoic acid	79-09-4	C_3_H_6_O_2_	74.1	1,640.7	1,697.680	1.27030
86	Linalyl acetate	115-95-7	C_12_H_20_O_2_	196.3	1,612.2	1,596.181	1.23750
87	Linalool oxide	60047-17-8	C_10_H_18_O_2_	170.3	1,463.8	1,158.358	1.80961
88	α-Thujone-M	546-80-5	C_10_H_16_O	152.2	1,454.8	1,136.202	1.37721
	α-Thujone-D	546-80-5	C_20_H_32_O_2_	304.5	1,455.1	1,136.895	1.87664
89	(E)-2-Octenal	2548-87-0	C_8_H_14_O	126.2	1,452.3	1,129.971	1.83015
90	Tetrahydrolinalool	78-69-3	C_10_H_22_O	158.3	1,432.6	1,082.889	1.26370
91	5-Methyl-2(3H)-furanone	591-12-8	C_5_H_6_O_2_	98.1	1,425.2	1,065.756	1.13555
92	2,3,5-Trimethylpyrazine	14667-55-1	C_7_H_10_N_2_	122.2	1,403.3	1,016.453	1.16155
93	3-Methyl-3-buten-1-ol	763-32-6	C_5_H_10_O	86.1	1,270.7	765.507	1.17519
94	p-Cymene	99-87-6	C_10_H_14_	134.2	1,273.2	769.369	1.73357
95	1,8-Cineole-M	470-82-6	C_10_H_18_O	154.3	1,222.2	693.519	1.30554
	1,8-Cineole-D	470-82-6	C_20_H_36_O_2_	308.6	1,222.5	693.940	1.72356
96	Limonene	138-86-3	C_10_H_16_	136.2	1,223.7	695.624	1.20719
97	1,4-Cineole	470-67-7	C_10_H_18_O	154.3	1,206.4	671.543	1.73531
98	1-Penten-3-ol	616-25-1	C_5_H_10_O	86.1	1,181.2	628.415	0.94307
99	2-Methyl-2-pentenal	623-36-9	C_6_H_10_O	98.1	1,175.3	615.854	1.15758
100	α-Phellandrene	99-83-2	C_10_H_16_	136.2	1,174.1	613.341	1.69846
101	3-Carene-M	13466-78-9	C_10_H_16_	136.2	1,149.8	564.352	1.20759
	3-Carene-D	13466-78-9	C_20_H_32_	272.4	1,149.6	563.933	1.67872
102	Sabinene-M	3387-41-5	C_10_H_16_	136.2	1,143.0	551.371	1.21549
	Sabinene-D	3387-41-5	C_20_H_32_	272.4	1,143.0	551.371	1.63924
103	Dimethyl disulfide	624-92-0	C_2_H_6_S_2_	94.2	1,101.9	478.933	1.13258
104	Methyl hexanoate	106-70-7	C_7_H_14_O_2_	130.2	1,191.2	650.353	1.27736
105	(E)-2-Butenal	123-73-9	C_4_H_6_O	70.1	1,076.6	444.012	1.20011
106	2-Ethylbutanal	97-96-1	C_6_H_12_O	100.2	1,023.9	381.384	1.21918
107	2-Butanol	78-92-2	C_4_H_10_O	74.1	1,027.0	384.813	1.16193
108	Ethyl isobutyrate	97-62-1	C_6_H_12_O_2_	116.2	1,001.2	357.197	1.57458
109	2-Ethylfuran	3208-16-0	C_6_H_8_O	96.1	986.4	344.644	1.04162
110	2,5-Dimethylfuran	625-86-5	C_6_H_8_O	96.1	961.6	326.442	1.37048
111	2-Methyl-2-propanol	75-65-0	C_4_H_10_O	74.1	950.2	318.440	1.32770

The suffixes M and D denote the monomer and dimer, respectively, of the same substance. MW: Molecular Weight. RI: Retention Index. Rt [sec]: Retention Time. Dt [RIPrel]: Drift Time (relative to RIP).

#### 3.1.3 GC-IMS fingerprint analysis

The construction of the fingerprint profiles reveals differences in the VOC signal profiles of *Artemisia argyi* Folium under varying irradiation doses (Figure 2). Each row in the fingerprint represents a sample, and each column represents the signal peak of a given VOC across different samples. The color brightness of each cell is positively correlated with the signal intensity of that compound in the corresponding sample: brighter colors (approaching bright yellow or white) indicate higher signal intensity, whereas darker colors (approaching deep blue or black) indicate lower signal intensity, or even no detection. The fingerprint provides complete VOC signal information for each sample and facilitates comparison of differences among samples.

**FIGURE 2:**
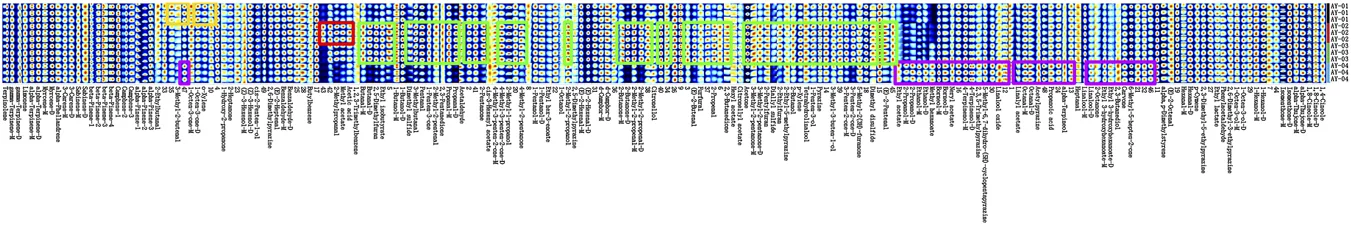
Fingerprint of headspace VOCs of hydrodistilled EO from *Artemisia argyi* Folium.

A comparative analysis of the VOC fingerprints of the four *A. argyi* Folium samples was conducted (as shown in Figure 2). The results reveal the following: In AY-01, compounds such as 3-Methyl-2-butenal, 1-Octen-3-one-D and o-Xylene show relatively high signal intensity, as indicated by the yellow box. In AY-02, compounds including 2-Methylpropanal, Methyl acetate, Acetic acid (E)-2-Pentenal, Tetrahydrolinalool, Dimethyl disulfide, 5-Methyl-2(3H)-furanone, 3-Methyl-3-buten-1-ol, Pyrazine and 2-Butanol exhibit elevated signal intensity, as shown by the red and green boxes. In AY-03, compounds such as (E)-2-Pentenal, Tetrahydrolinalool, Dimethyl disulfide, 5-Methyl-2(3H)-furanone, 3-Methyl-3-buten-1-ol, Pyrazine, 2-Butanol, Ethyl isobutyrate, 2, 3-Pentanedione, 4-Methyl-2-pentanone and 2-Propenal are present higher signal intensity, as indicated by the green box. In AY-04, compounds including 6-Methyl-5-hepten-2-one, Carvone, 2, 3-Butanediol, Ethyl 3-hydroxyhexanoate-D, Ethyl 3-hydroxyhexanoate-M, 2-Octanone, Linalool-D, α-Terpineol, Propanoic acid, Acetylpyrazine, Octanal-D and Octanal-M show increased signal intensity, as shown by the purple box.

### 3.2 Statistical analysis of GC-IMS

#### 3.2.1 Principal component analysis (PCA)

By analyzing complex data from multiple sample groups, PCA can reveal similarities and dissimilarities among samples (Souza et al., 2024). Generally, shorter distances between sample points reflect higher measurement repeatability, whereas larger distances imply greater variation. In the score plot, the degree of clustering among biological replicates within each group reflects the reproducibility of the measurements, while the separation distance between different groups indicates the extent of chemical compositional differences induced by the treatments. The 2D PCA score plot (Figure 3) was generated using data from three independently irradiated and extracted biological replicates (n = 3) for each dose group, as described in Sections 2.2–2.4. Regarding intergroup distribution, the distance between groups AY-02 and AY-03 is relatively small, suggesting similar VOC signal patterns in *Artemisia argyi* Folium treated with 3.0 kGy and 6.0 kGy of ^60^Co irradiation. Groups AY-01 and AY-04 from separate clusters, indicating differences in VOC signal patterns and suggesting that varying intensities affect *A. argyi* Folium VOCs differently. The close clustering within each group supports the reproducible treatment-related differences, rather than merely analytical repeatability. Nevertheless, these grouping tendencies should be interpreted with caution. Although independent biological replicates were used for each treatment, the number of biological replicates was limited (n = 3). Therefore, this multivariate analysis serves only as an observational exploratory tool and cannot provide definitive evidence for the physicochemical changes of volatiles caused by irradiation.

**FIGURE 3:**
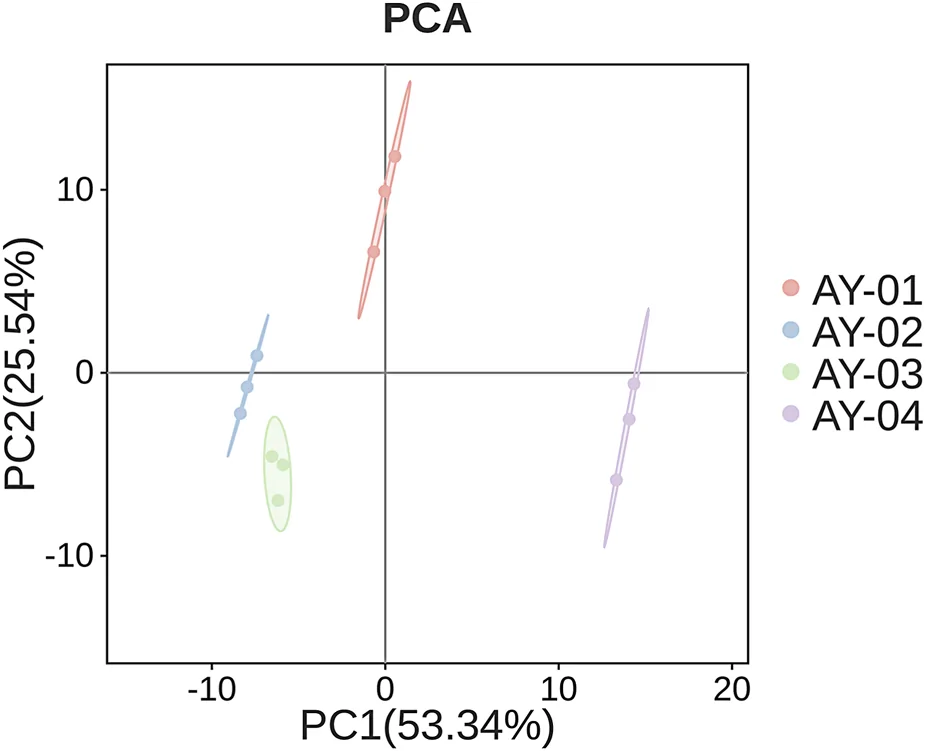
PCA Score Plot of headspace VOCs of hydrodistilled EO from *Artemisia argyi* Folium. Each data point represents an independently irradiated and extracted biological replicate (n = 3 per group).

#### 3.2.2 Cluster analysis (CA)

CA was performed for exploratory visualization of the volatile profiles across the four treatment groups (Figure 4). The heatmap color intensity reflects the relative signal response of a VOC in a sample: deeper pink indicates higher than average signal intensity, while deeper blue indicates lower than average signal intensity. As shown in Figure 4, AY-02 and AY-03 exhibited similar volatile patterns and clustered closely, whereas AY-01 and AY-04 displayed relatively independent branching characteristics. Compared to other groups, sample AY-01 presented lower signal intensity of several VOCs, including Myrcene-D, α-Terpinene-D, α-Phellandrene, β-Pinene-4, Ethyl hex-3-enoate, 1-Octen-3-one-D, 2-Ethylbutanal, 1-Pentanol-M, α-Pinene-1 and d-Camphor-M. In AY-04, relatively high signal intensities of 1,8-Cineole-M, Hexanal-D, 3-Carene-M, 6-Methyl-5-hepten-2-one, β-Pinene-2, Hexanal-M, 2-Propanol-M, 1-Octen-3-ol-D, α-Pinene-2, Ethanol-D, p-Cymene were detected. AY-02 and AY-03 shared highly similar volatile signal profiles, characterized by higher signal intensities of Propanal-M, Pentanal, 1-Penten-3-ol, Dimethyl disulfide, 4-Methyl-3-penten-2-one-D, Ethyl isobutyrate, Dimethyl sulfide, Acetaldehyde, 2-Methyl-1-propanol, Methyl acetate and Diallyl sulfide.

**FIGURE 4:**
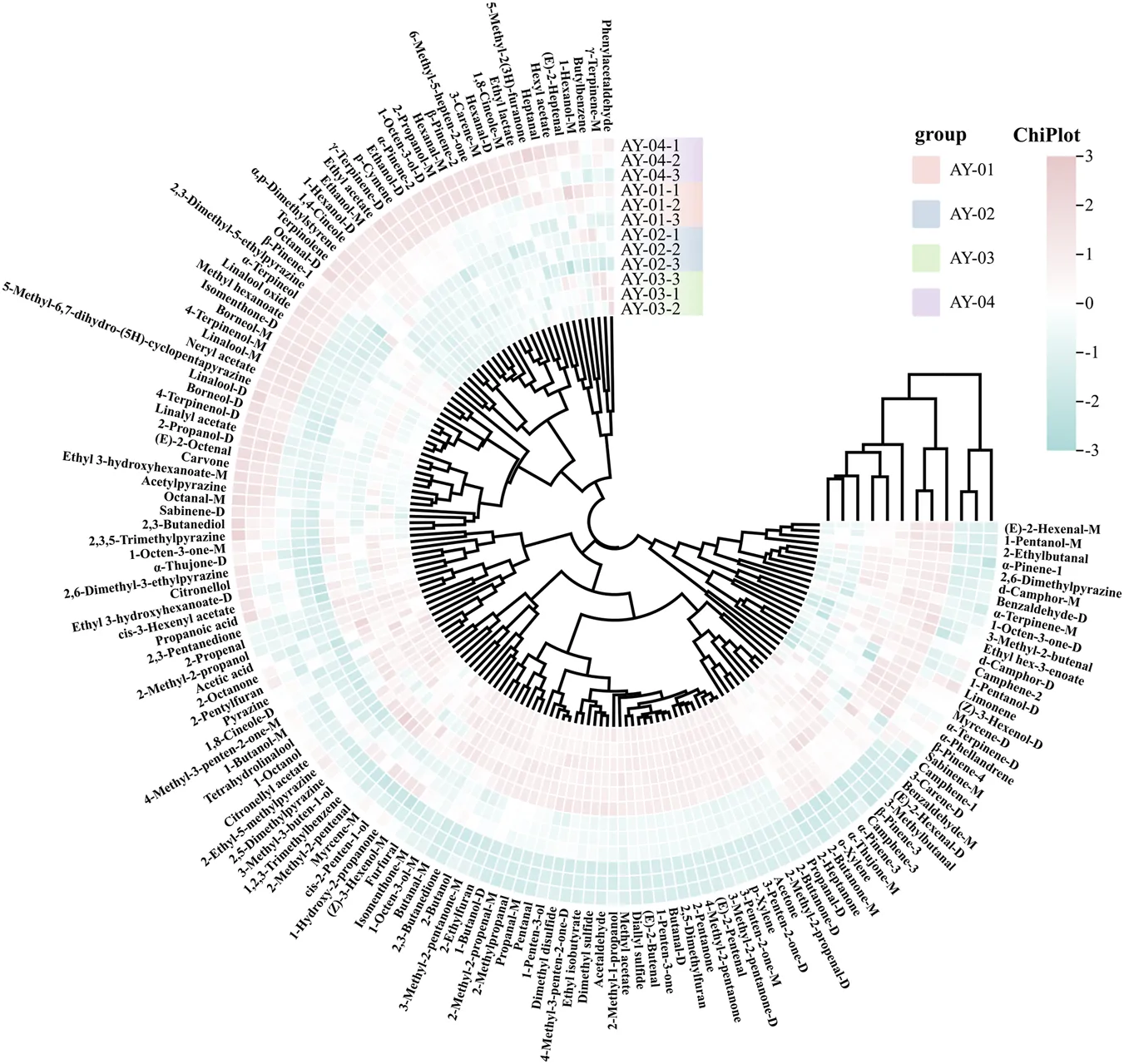
Cluster Heatmap of headspace VOCs of hydrodistilled EO from *Artemisia argyi* Folium Subjected to Four Different ^60^Co Irradiation Doses.

#### 3.2.3 Partial least-squares discriminant analysis (PLS-DA)

Discriminant analysis (DA), particularly PLS-DA, is a supervised classification method frequently employed in volatilomic or chemometric studies. It simplifies data by retaining variables that best explain intergroup differences, enabling effective discrimination among sample groups (Geng et al., 2024). The data from the four sample groups were imported into SIMCA software, and the resulting plot is depicted in Figure 5A. The sample distribution exhibited observational grouping trends consistent with the PCA results. Samples AY-02 and AY-03 are closely positioned, while AY-01 and AY-04 each forms independent clustering regions with minimal overlap, suggesting potential similarities in VOC profiles between AY-02 and AY-03, as well as distinguishable volatile patterns for AY-01 and AY-04. This conclusion aligns with the PCA plot. 200-fold cross-validation was performed to calculate the R^2^Y (0.993) and Q^2^ (0.888) values for model evaluation. A separate permutation test (200 permutations, 5 components) was conducted to assess model overfitting, as shown in Figure 5B. In this permutation test, all permutated models exhibited lower R^2^ and Q^2^ values than the original model at the abscissa of 1.0, and the regression line of Q^2^ intersects the abscissa at a negative value, preliminarily indicating that the established PLS-DA model has no obvious overfitting. To quantify each VOC’s contribution to the group discrimination model, Variable Importance in Projection (VIP) was calculated based on the PLS-DA model. Using VIP >1 as the threshold, VOCs with potential discriminatory power for sample classification were tentatively identified (Figure 5C). Major differential compounds include d-Camphor-D, γ-Terpinene-M, Benzaldehyde-D, Camphene-2, 1,2,3-Trimethylbenzene, 1-Octen-3-one-M, d-Camphor-M, Acetic acid, 2,3,5-Trimethylpyrazine, 2,6-Dimethylpyrazine, α-Terpinene-D, Myrcene-D, 2-Ethylbutanal, α-Pinene-3, Pyrazine, Butylbenzene, Carvone, 2-Propanol-D, cis-3-Hexenyl acetate, 2,3-Butanediol, 2,6-Dimethyl-3-ethylpyrazine, and Acetylpyrazine. These compounds may potentially serve as candidate indicators for exploring the effects of different irradiation doses on the VOC profiles of *Artemisia argyi* Folium.

**FIGURE 5:**
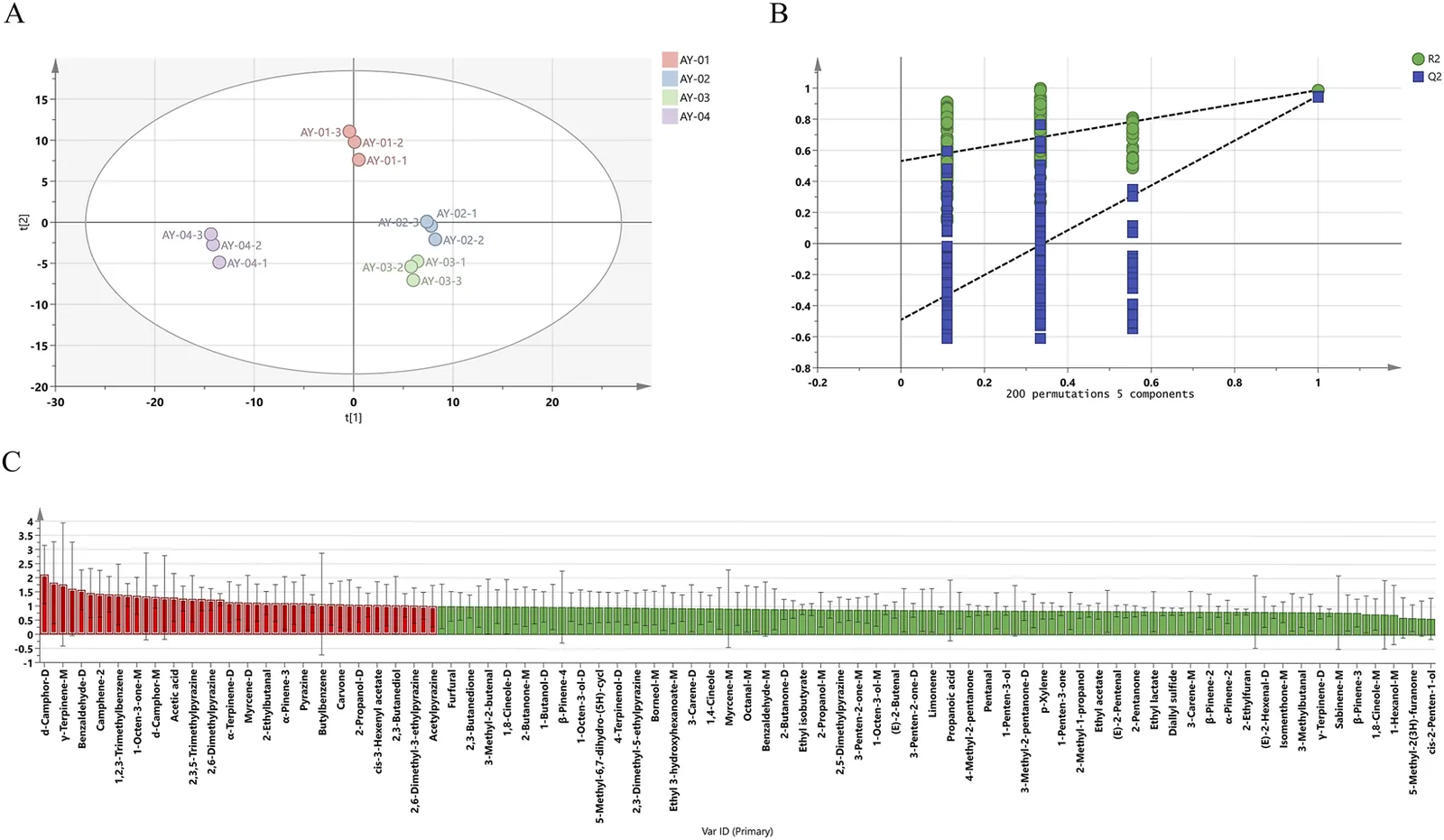
PLS-DA analysis of headspace VOCs of hydrodistilled EO from *Artemisia argyi* Folium under four different ^60^Co irradiation doses **(A)**; Permutation test of headspace VOCs of hydrodistilled EO from *Artemisia argyi* Folium under four different ^60^Co irradiation doses **(B)**; VIP values of headspace VOCs of hydrodistilled EO from *Artemisia argyi* Folium under four different ^60^Co irradiation doses **(C)**.

### 3.3 Effects of different irradiation doses on the antibacterial activity of *Artemisia argyi* Folium

#### 3.3.1 Determination of antibacterial activity

The inhibition zones of different EOs to *E. coli* and MRSA are shown in Figure 6. Samples AY-01 to AY-04 displayed inhibitory effects against both *E. coli* and MRSA. Among them, AY-03 produced the largest inhibition zone diameter (16.0 ± 0.1 mm) against *E. coli*, while AY-02 yielded the largest inhibition zone diameter (16.1 ± 0.1 mm) against MRSA.

**FIGURE 6:**
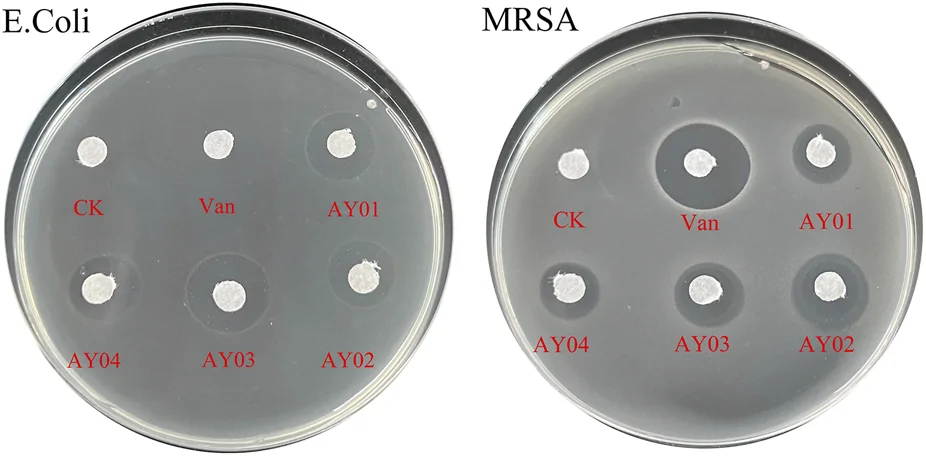
The inhibition zone of AY-01-AY-04 to *Escherichia coli* (left) and MRSA (right). Note: CK: distilled water; Kan: Kanamycin sulfate (positive control for *Escherichia coli* and MRSA); Van: Vancomycin (positive control only for MRSA).

The MIC and MBC were tested by diluting the samples of AY-01---AY-04 to concentrations of 10% and 0.078%; against *E. coli*, the MIC and MBC of AY-01 were 0.4% and 1.25%, respectively, while the MIC and MBC values for AY-02, AY-03, and AY-04 were all 0.2% and 1.25%. Against MRSA, at concentrations of 10% and 0.078%, the MIC and MBC of AY-01 and AY-02 were both 0.2% and 0.625%, those of AY-03 and AY-04 were both 0.1% and 0.156%.

The inhibition rates of AY-01---AY-04 against *E. coli* and MRSA at concentrations ranging from 4‰ to 0.0015‰ are presented in Figure 7. The calculated IC_50_ values of AY-01---AY-04 against *E. coli* were 1.095‰, 0.054‰, 0.169‰, and 0.047‰, respectively, while against MRSA, the IC_50_ values were 0.118‰, 0.217‰, 0.284‰, and 0.246‰, respectively.

**FIGURE 7:**
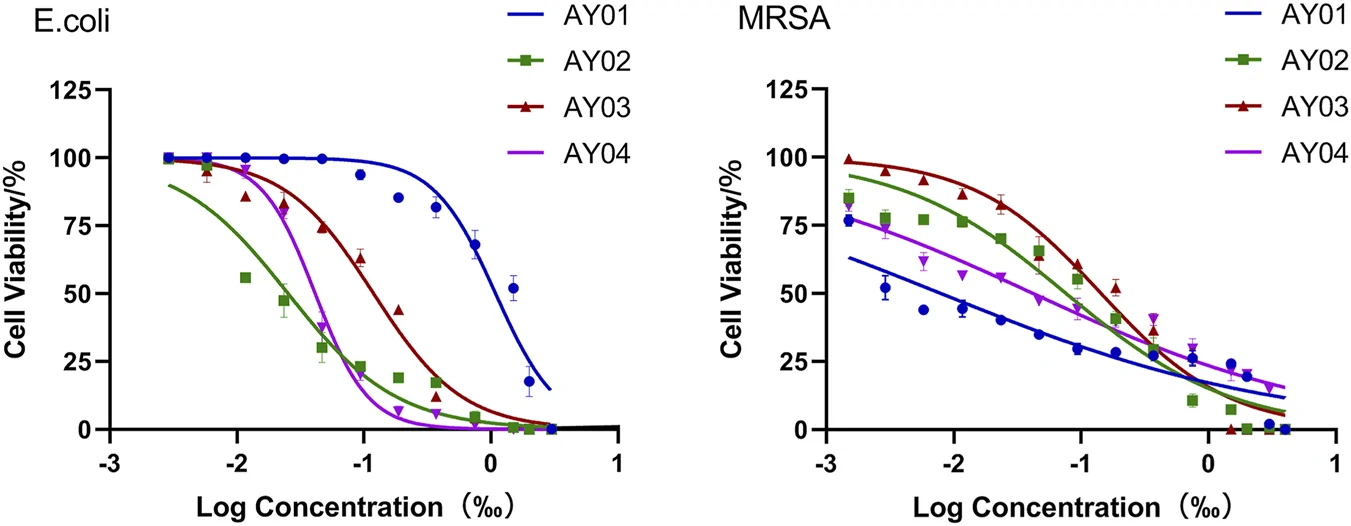
The inhibition rates of AY-01-AY-04 against *Escherichia coli* (left) and MRSA (right).

### 3.4 Pearson correlation analysis

Pearson correlation analysis was utilized to ascertain the magnitude and direction of the linear relationships between two continuous variables, with coefficient values ranging from −1 to 1. The analysis included significance tests to establish whether the observed correlations were statistically significant. This method was employed to evaluate the relationships between differentially expressed markers (VIP ≥1) of *Artemisia argyi* Folium under various irradiation doses and their IC_50_ values against *E. coli* and MRSA. IC_50_ represents the concentration of a compound required to inhibit bacterial growth by 50%, with lower values indicating stronger antibacterial activity. A negative correlation (r < 0) indicates that higher relative signal intensity of the compound is associated with a lower IC_50_ value of the whole oil. This suggests a potential contribution of this compound to the antibacterial activity of the oil, but does not establish causation. Further studies with purified compounds are needed to confirm the direct antibacterial effects. While a positive correlation (r > 0) suggests that higher relative signal intensity of the compound is associated with a higher IC_50_ value of the whole oil, indicating a less favorable contribution to antibacterial activity in the context of the whole oil. As depicted in Figure 8, pink denotes a positive correlation, and blue signifies a negative correlation. α-Phellandrene had a strong negative correlation with the IC_50_ against MRSA (r = −0.99, p < 0.05), while Pyrazine had a positive correlation with the IC_50_ against MRSA (r = 0.96, p < 0.05). 1-Octen-3-one-M (r = −0.95, p < 0.05), β-pinene-1 (r = −0.95, p < 0.05), and α-thujone-D (r = −0.99, p < 0.05) all had negative correlations with the IC_50_ against *E. coli*. Cis-3-hexenyl acetate (r = −0.99, p < 0.01) had an extremely strong negative correlation with the IC_50_ against *E. coli*. It should be noted that such correlations only indicate covariation and do not confirm causal effects of individual compounds.

**FIGURE 8:**
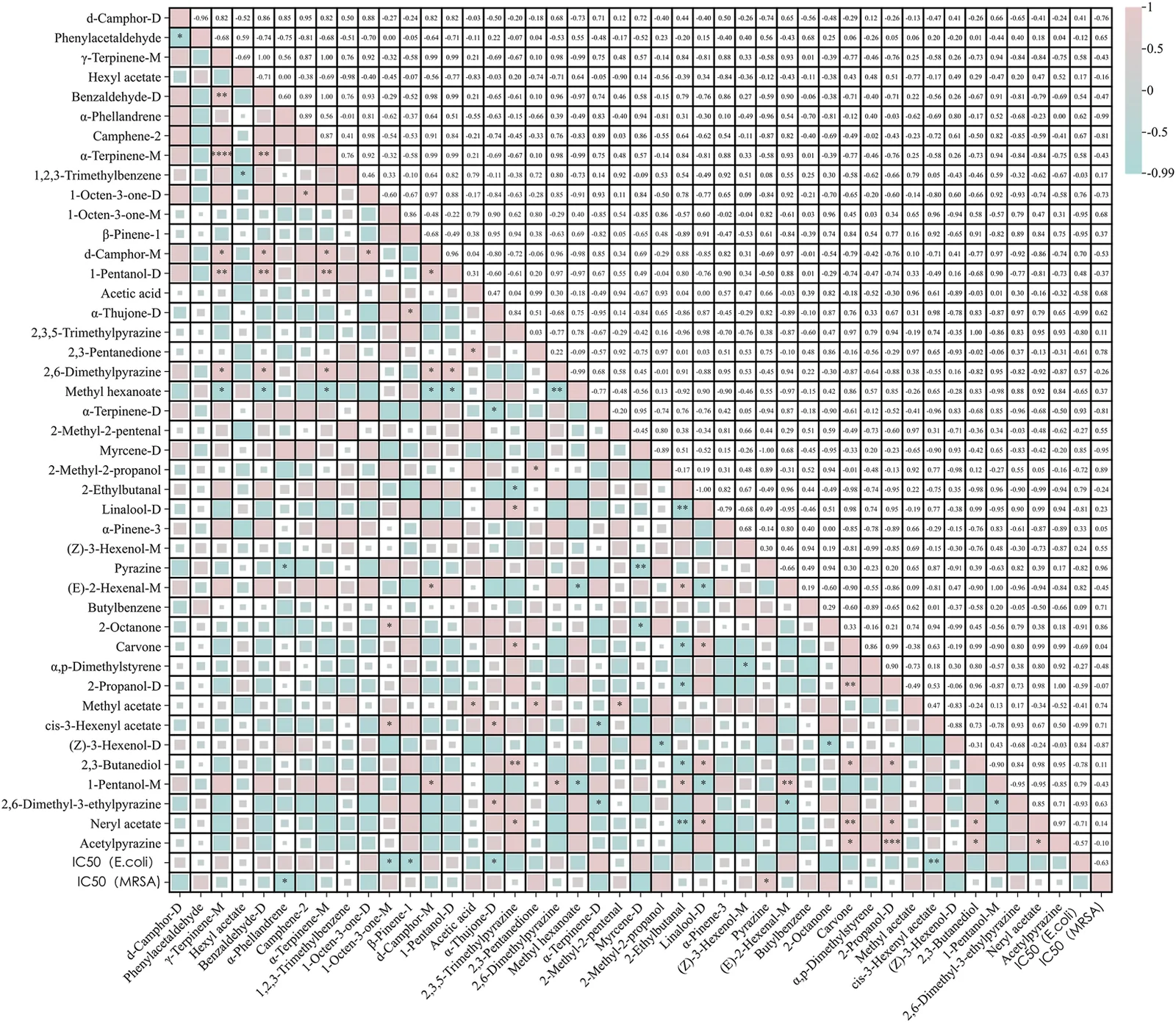
Pearson correlation analysis between differential headspace VOCs markers (VIP >1) released from *Artemisia argyi* Folium under different irradiation doses and their IC_50_ values against *Escherichia coli* and MRSA (*p < 0.05, **p < 0.01, ***p < 0.001, ****p < 0.0001).

## 4 Discussion

This study employed GC-IMS and multivariate statistical analysis to systematically investigate the dose-dependent effects of ^60^Co irradiation (0–9 kGy) on the volatile profiles and antibacterial properties of *Artemisia argyi* Folium EO. As observed in our earlier work on irradiated herbal materials, VOC composition barely changed under low-to-moderate irradiation (3–6 kGy). In contrast, marked remodelling of volatile compounds was observed at 9 kGy, showing that the substantial VOC compositional shift takes place within the tested dose interval from 6 to 9 kGy. Such dose-dependent responses of volatile constituents vary across aromatic plant species. Previous studies have reported that the chemical composition of *Cnidii Fructus* remained stable at 3 kGy, pronounced compositional changes occurred at 6 kGy, and substantial chemical composition reconstruction was observed at 9 kGy. While a significant decrease of *Satureja mutica* was detected in components with 7.5 and 10 kGy irradiation (Huang et al., 2026; Navehsi et al., 2024). It further confirmed that plant species strongly determine the radiation sensitivity of herbal volatile compounds. From a practical processing perspective, the observed VOC changes indicate that doses within 3–6 kGy are favourable to preserve the volatile composition of hydrodistilled EO from *A. argyi* Folium during decontamination.

The VOC variations induced by ^60^Co irradiation are hypothesized to arise from physical radiolytic reactions rather than biological metabolism, since dried medicinal materials contain no viable cells. High-dose irradiation significantly increased the relative abundance of oxygenated terpenoids, including Linalool, α-Terpineol and Carvone. Such compound transformation may align with classical radiolytic mechanism, in which ^60^Co radiation cleaves monoterpene hydrocarbons and promotes the generation of oxygenated volatile derivatives (Belcadi et al., 2023). We speculate that radiation-induced chemical rearrangement could be a core contributor VOC profile divergence in high-dose groups.

^60^Co irradiation effectively improved the antibacterial performance of *A. argyi* EO, but the promotional effects varied significantly with bacterial strains and detection methods. In general, irradiation enhanced the inhibitory activity against *E. coli*, while its influence on MRSA was dose- and index-dependent. The inconsistent optimal doses and divergent activity trends across disc-diffusion, MIC/MBC and IC_50_ assays are well-recognized methodological caveats for EO testing. Diffusion-based and broth dilution assays differ substantially in the phase behavior and bioavailability of volatile lipophilic compounds (Hulankova, 2024). Moreover, the heterogeneous antibacterial responses between *E. coli* and MRSA observed in this study are in line with previous findings on irradiated ylang-ylang EO. These earlier studies confirmed that ^60^Co irradiation produces strain-specific antibacterial variations rather than universal activity changes (El-Sayed et al., 2023). Combined with reports on *Tanacetum annuum* EO (Belcadi et al., 2023), the present results verify that radiation-mediated VOC modification inevitably reshapes the biological activity of plant EOs in a strain-dependent manner.

Correlation analysis further clarified the material basis for antibacterial differences. The strong negative correlation between α-phellandrene abundance and MRSA IC_50_ indicated that α-phellandrene could serve as a candidate marker associated with the antibacterial activity against MRSA, consistent with previously reported antibacterial characteristics of this monoterpene (Dam et al., 2025). In contrast, pyrazine was also identified as a candidate marker correlated with MRSA inhibition. For *E. coli*, monoterpenes such as β-pinene and α-thujone-D can damage bacterial membrane integrity and cause intracellular substance leakage (Melkina et al., 2021; Nogueira et al., 2021), which reasonably explains the improved anti-*E. coli* performance of irradiated *A. argyi* EO.

This study demonstrates that GC-IMS is a reliable technique for characterizing subtle radiation-induced volatile changes in *A. argyi*. The combination of GC-IMS fingerprinting and multivariate statistics provides an effective strategy for distinguishing irradiation doses and screening bioactive volatile markers.

Several limitations of this study should be acknowledged. First, although hydrodistillation may cause minor thermal modification of volatiles, uniform extraction conditions across all samples guaranteed the comparability among groups. Headspace GC-IMS only detects high-vapor-pressure fractions of distilled EO; hence VOC data represent headspace accessible compounds rather than *in-situ* volatiles of irradiated raw leaves. Second, GC-IMS analysis provides semi-quantitative relative intensity rather than absolute content. Third, this study focused on chemical and antibacterial variations induced by irradiation, so further studies are required to explore the relationships among irradiation dose, microbial inactivation efficiency and volatile composition. In addition, variable antibacterial results from different detection platforms suggest that multiple evaluation systems and expanded pathogen strains are necessary for comprehensive assessment of irradiation-modified herbal EO activity. Fourth, direct evidence supporting monoterpene cleavage and radiation-triggered chemical rearrangement was not obtained in this work. The proposed radiolytic mechanism is therefore speculative and awaits further experimental confirmation.

## 5 Conclusion

This study compared the effects of different ^60^Co irradiation doses on the VOC profiles and antibacterial activity of *A. argyi* Folium EO. GC-IMS demonstrated that 3.0 and 6.0 kGy preserved similar volatile composition of hydrodistilled EO, while 9.0 kGy triggered substantial VOC alterations; irradiation produced strain-dependent antibacterial effects. Agar diffusion assays revealed the largest inhibition zone against *E. coli* in the 6.0 kGy group and the maximum zone against MRSA in the 3.0 kGy group. MIC, MBC, and IC_50_ analyses further reflected the dose-dependent antibacterial variations of irradiated EO. These results illustrate dose-related chemical and functional changes and provide guidance for selecting suitable irradiation doses during *A. argyi* Folium processing.

## Data Availability

The original contributions presented in the study are included in the article/Supplementary Material, further inquiries can be directed to the corresponding authors.
